# Diagnostic performance of CareStart™ malaria HRP2/pLDH test in comparison with standard microscopy for detection of uncomplicated malaria infection among symptomatic patients, Eastern Coast of Tanzania

**DOI:** 10.1186/s12936-019-2990-9

**Published:** 2019-11-05

**Authors:** George M. Bwire, Billy Ngasala, Manase Kilonzi, Wigilya P. Mikomangwa, Fatuma F. Felician, Appolinary A. R. Kamuhabwa

**Affiliations:** 1 Department of Pharmaceutical Microbiology, Muhimbili University of Health and Allied Sciences, P.O. Box 65013, Dar es Salaam, Tanzania; 2 Department of Parasitology and Medical Entomology, Muhimbili University of Health and Allied Sciences, P.O. Box 65001, Dar es Salaam, Tanzania; 3 Department of Clinical Pharmacy and Pharmacology, Muhimbili University of Health and Allied Sciences, P.O. Box 65013, Dar es Salaam, Tanzania

**Keywords:** CareStart™ malaria, Microscopy, Diagnostic performance, Kibiti, Tanzania

## Abstract

**Background:**

CareStart™ malaria HRP2/pLDH (Pf/pan) combo test is one of the several rapid diagnostic tests (RDT) approved for diagnosis of malaria at the point of care in Tanzania. However, there are limited studies on the diagnostic performance of RDT after wide scale use in primary health care facilities in Tanzania. Therefore, this study was carried out to determine the diagnostic performance of RDT when compared with blood smear (BS) microscopy as a reference standard.

**Methods:**

A cross-sectional study was conducted between March and August 2019 at Kibiti Health Centre, Pwani region, Tanzania. Blood samples for malaria tests were collected from patients with malaria symptoms. Diagnostic performance parameters of RDT, i.e. sensitivity, specificity, positive and negative likelihood ratios (LR+/−), diagnostic accuracy and predictive values were determined using contingency table. An agreement between RDT and microscopy was statistically determined by Cohen’s kappa test.

**Results:**

Of 980 patients screened, 567 (57.9%) were found to be malaria positive by RDT, whereas 510 patients (52%) were positive by microscopy. Of the 510 microscopy-positive patients, 487 (95.5%) were infected with *Plasmodium falciparum*. The geometric mean parasite density was 2921parasites/µl, whereas majority (68.6%) of patients had parasite density greater than 10,000/µl. The sensitivity, specificity, positive and negative predictive values of CareStart™ were 99.8%, 87.6%, 89.8%, and 99.8%, respectively. The LR+ and LR− were 8.0 and 0.002, respectively. The diagnostic accuracy was 0.5. There was a strong agreement between the results obtained using CareStart™ and BS microscopy (kappa = 0.863, P < 0.0001).

**Conclusion:**

CareStart™ malaria HRP2/pLDH (Pf/pan) had high sensitivity and strong agreement with microscopy results. However, moderate specificity of RDT resulted in a substantial number of patients with false positive malaria test. Wherever available, microscopy should be used to confirm RDT test results.

## Background

Global malaria control and elimination programme advocates test and treat as one of the key strategies toward its achievements [1, 2]. In Tanzania, microscopy is still considered as a reference standard in malaria diagnosis at the point of care [3]. However, routine use of microscopy for accurate diagnosis of malaria in the health facilities faces a number of challenges. These challenges include qualified human resources, stable electrical power supply, quality of blood slides, low parasite densities, and altered parasite morphology caused by chemoprophylaxis or empiric therapy [3–6].

Microscopic examination involves the quantification of malaria parasites against white blood cells (WBCs) or red blood cells (RBCs) [7, 8]. To quantify malaria parasites against RBCs, the parasitized RBCs among 500–2000 RBCs are quantified on the thin smear and results expressed as percentage parasitaemia. On the other hand, quantification of malaria parasites against WBCs are tailed against WBCs until 500 parasites or 1000 WBCs are counted, then express as parasites per microlitre of blood [6–8]. Rapid diagnostic test **(**RDT) is mostly used in countries with limited resources for diagnosis of malaria due its advantages in these settings. The advantages of RDT include easy to use, provision of results from whole blood within 20 min, easy to carry diagnostic kits and require less skilled personnel [9, 10].

Diagnosis using RDT are made to target different antigens, such as histidine-rich protein-2 (PfHPR2) for *Plasmodium falciparum*, *Plasmodium* lactate dehydrogenase (pLDH) and aldolase, which are common to all human *Plasmodium* species [11]. Nevertheless, studies have reported variations in the accuracy for diagnosis of malaria when using RDT. For instance, diagnosis of malaria using RDT with PfHRP2 was reported to have slightly better sensitivity than *P. falciparum* lactate dehydrogenase (Pf-pLDH) detecting RDT [11–13].

Moreover, PfHRP2 tests have several other limitations, including persistence of the PfHRP2 antigens and false-positive reactions due to other infectious agents or immunological factors [12]. In addition, findings from studies conducted in Rwanda [14] and Senegal [15] reported undetected cases with deletion of PfHRP2. However, blood smear microscopy (BS microscopy) remains to be the reference standard method for malaria diagnosis and monitoring of the treatment progression especially in malaria endemic regions [3, 6–8, 16]. Therefore, the current study aimed to evaluate the diagnostic performance of CareStart™ malaria HRP2/pLDH (Pf/pan) combo and its agreement to BS microscopy for diagnosis of malaria among clinically suspected malaria patients following the recent wide scale use of RDT for diagnosis of malaria in primary health centres in Tanzania.

## Methods

### Study area

This study was carried out at Kibiti Health Centre (KHC) situated in Kibiti District, Pwani region, Eastern Coast, Tanzania. Kibiti is one of the 8 districts of the Pwani Region of Tanzania. It is bordered to the North by the Kisarawe and Mkuranga Districts, to the East by the Indian Ocean, to the South by the Lindi Region and to the West by the Morogoro Region. The region has malaria cases through out the year with the approximated prevalence of 5.3%, where *P. falciparum* is responsible to more than 95% of all malaria cases [17]. Currently, KHC as a district hospital attends both in-patient and outpatients where patients suspected with malaria are subjected to RDT testing then if found positive malaria management is initiated [18].

### Study design

This was a cross sectional study conducted between March and August 2019 with the aim of determining the diagnostic performance of RDT test compared to the blood smear (BS) microscopy as a standard method among patients of all ages with suspected malaria infection. Diagnostic accuracy, sensitivity, specificity, likelihood ratio (LR), and predictive values were calculated as described by Šimundić et al. [19].

### Study population

Patients with age above 6 months attending clinic at KHC who presented with symptoms suggestive of malaria infection were recruited in the study. The symptoms such as fever, general body weakness and headache were confirmed by the attending physician [20]. In addition, the attending physician interviewed patients on the regular use of insecticide-treated bed nets using Yes/No question. After physical examination, patients were directed to provide blood samples for diagnosis of malaria. Patients with severe malaria and children with malnutrition were excluded from the study.

### Sample size and sampling technique

Nine hundred and eighty (980) participants were recruited to participate in this study. The sample size was calculated using single population proportion formula considering 95% confidence interval (CI) and proportion (P) of 5.7% [17] as follows; *n* = Z_α/2_P (1 − P)/ε^2^ where n is sample size, Z_α/2_P = 1.96 for 95% confidence level, ε is the marginal error. All the recruited study participants were patients presenting with symptoms suggestive of malaria infection as determined by their attending clinicians. All malaria suspected patients between March and August 2019 were eligible to participate in the study.

### Clinical assessment and laboratory procedures

#### Determination of body temperature

The armpit body temperature was measured using clinical digital thermometer as per manufacturer’s specifications (IndiaMART, India). The body temperature of < 37.5 °C and ≥ 37.5 °C were classified as normal and febrile fever, respectively [21].

#### Determination of haemoglobin levels

Haemoglobin (Hb) level was measured photometrically by Hemocue Hb 201^+^ (Angelholm, Sweden) using microcuvette following manufacturer’s instructions. The drop of blood was collected in Hb 201 microcuvette and read using HemoCue Hb 201 + device and results were recorded in g/dl. The Hb level of < 6 g/dl, 6–11.5 g/dl and > 11.5 g/dl were classified as anaemia, mild and normal, respectively, as previously described [22].

### Diagnosis of malaria by CareStart™ malaria HRP2/pLDH (Pf/pan) Combo test

Diagnosis of malaria by rapid test was done according to manufacturer’s instructions using CareStart™ malaria (Lot 05EDE011A, Access Bio, Ethiopia) stored at temperature between 2 °C and 30 °C. Briefly, two laboratory technicians recorded the results independently during the daylight, assisted by a standard electric bulb. The laboratory technicians were blinded to each other’s readings and to the results of microscopy. The discrepancies were resolved using a third reader.

#### Diagnosis of malaria by microscopy

Duplicate thin and thick blood films were prepared. A total of 980 patients, i.e. both CareStart™ malaria positive and negative individuals were subjected to BS microscopy. Slides containing thin and thick smears were air-dried for 30 min at room temperature followed by staining with 5% Giemsa (GIBCO, Scotland, UK) at pH 7.2. After staining, the slides were examined by two independent experienced microscopists (undergone national or regional training and certified to perform malaria testing by Tanzania Ministry of Health) using light microscopy with 1000 × oil- immersion lenses. Briefly, malaria parasites count was recorded until 200 leukocytes were counted. If less than 10 parasites were found, counting was extended to 500 leukocytes [23]. Parasite densities were calculated by assuming a total leukocyte count of 8000/μl (17). For quality control, 100 blood slides were randomly selected and observed by a senior microscopist at Muhimbili University of Health and Allied Sciences. In addition, a senior microscopist was also used to resolve discrepancies between the readers.

### Data management and analysis

Information recorded in the data collection sheet were entered in Microsoft Excel sheet (Redmond, WA) and exported to Prism 7 software (GraphPad Software, USA) for determining the CareStart™ malaria diagnostic accuracy. Sensitivity, specificity, LR and predictive values were obtained using two-by-two cross table. Agreement between CareStart™ and BS microscopy was determined by Cohen’s kappa test using statistical package for social sciences (SPSS version 25 software, Chicago Inc., USA). The value of kappa, < 0.59, 0.6–0.79, 0.8–0.9 and > 0.9 were considered weak, moderate, strong and almost perfect agreement, respectively [24]. Likelihood ratios for positive (LR+) and negative (LR)-test results were considered good when LR+ was > 10, and LR− < 0.1. The diagnostic accuracy was explored from receiver-operating characteristics (ROC) curve. Using the area under the curve (AUC) for determination of diagnostic accuracy; 0.9–1.0, 0.8–0.9, 0.7–0.8, 0.6–0.7, 0.5–0.6, and < 0.5 were considered equivalent to excellent, very good, good, sufficient, bad and test not useful, respectively [18, 22]. The *P* value for significance was considered at < 0.05.

### Ethical consideration

Ethical approval (Reference number DA.282/298/01A.C/) was obtained from Muhimbili University of Health and Allied Sciences Institutional Review Board. In addition, the National Institute for Medical Research also provided ethical clearance (Reference number NIMR/HQ/R.8A/Vol.IX/3107) for this study. Written informed consent was obtained from each study participant and from parent/legal guardian for children. Furthermore, verbal assent was obtained from children with age above 5 years before requesting consent from their parents/legal guardian. Permission to conduct the study at KHC was obtained from both Kibiti District Medical Officer and KHC Medical officer in-charge. Consented patients were requested to give blood spots from a finger prick for RDT, thick and thin blood smears for microscopy. Patients found positive for malaria by microscopy and those with low Hb levels were immediately communicated to their attending physician for further management. Names and other personal details were not disclosed for confidentiality purposes.

## Results

### Participants’ recruitment flow chart

**Fig. 1:**
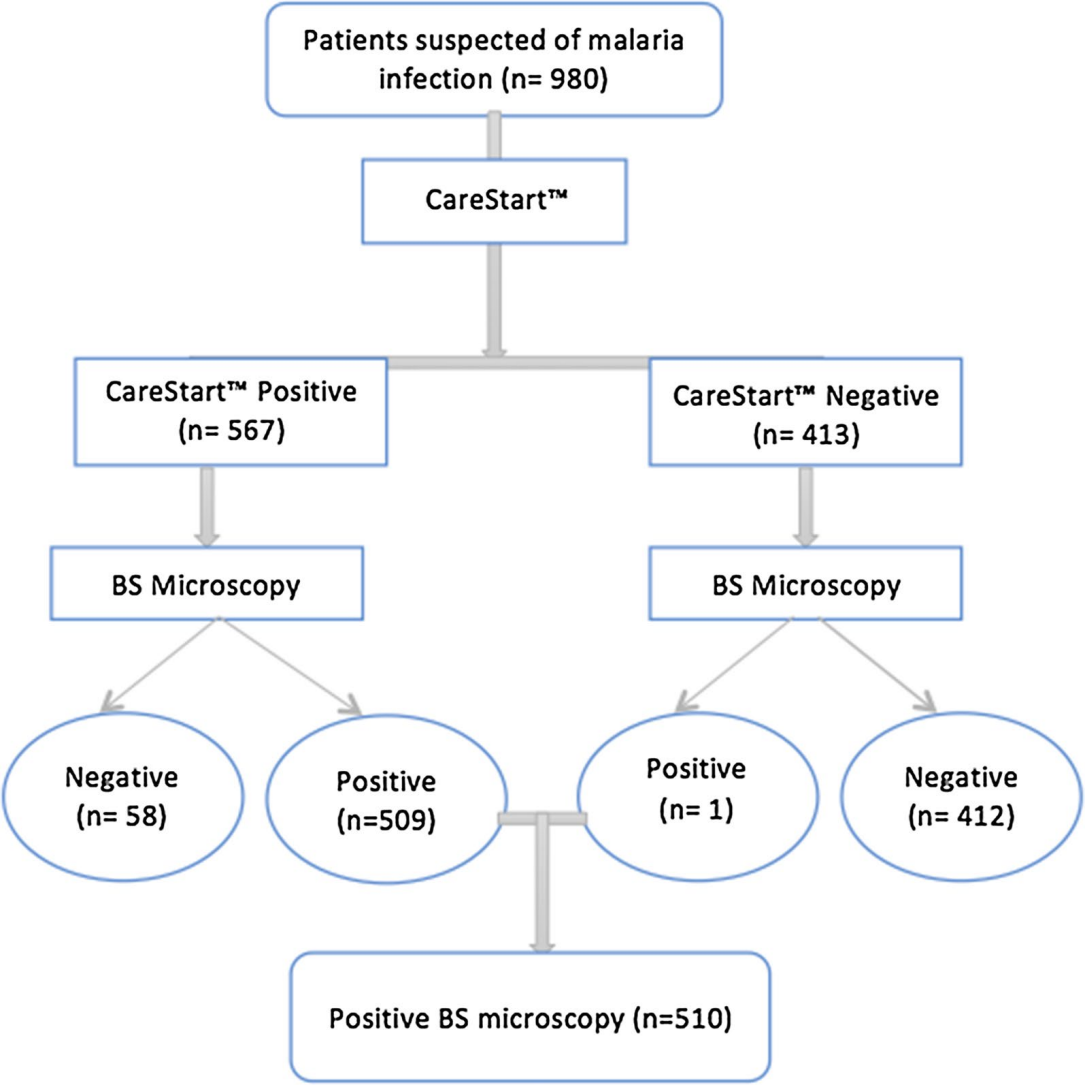
Study participants’ malaria diagnosis flow chart (n = number of cases)

A total of 980 were clinically suspected to have malaria infection. Of 980 patients, 567 (57.9%) were found to be malaria positive as determined by CareStart™, while 413 patients were malaria negative. Both CareStart™ malaria positive and negative individuals were subjected to BS microscopy. Out of the 413 patients who were malaria negative by CareStart™, one patient (0.2%) was confirmed positive by BS microscopy. Therefore, out of 980 individuals who were suspected to have malaria infection based on presenting symptoms, only 510 patients (52%) were confirmed by microscopy to be malaria positive (Fig. 1).

### General characteristics of patients

Out of the 980 participants who were recruited to participate in the study, 529 (54%) were females. The median [Interquartile Range (IQR)] age was 6 (3–18.5) years whereas 394 (40.1%) participants had age below 5 years. A total of 894 participants (91.3%) reported the use of insecticide-treated bed nets. The mean [(Standard deviation (± SD)] haemoglobin level was 10.5 (1.7) g/dl and 776 (79.2%) participants had mild anaemia. A majority (65.6%) of the participants had fever. Of the 510 BS positive patients, 487 (95.5%) were *P. falciparum* mono-infected. The rest 13 (4.6%) patients were infected with other species of *Plasmodium*. The geometric mean parasite density was 2921 parasites/μl, whereas the majority (68.6%) of patients had parasite density of above 10,000/µl (Table 1).

**Table 1:** Participants’ and malaria parasites characteristics (n = number of cases)

Characteristics	n (%)
Gender	
Male	451 (46)
Female	529 (54)
Median age (years)	
Median (IQR)	6 (3–18.5)
Age group (years)	
< 5	394 (40.1)
5–18	343 (35)
> 18	244 (24.9)
Pregnant women	74 (7.5)
Use of insecticide-treated bed nets	
Yes	894 (91.3)
No	86 (8.7)
Mean Hb level (g/dl)	
Mean ± SD	10.5 ± 1.7
Hb level distribution (g/dl)	
< 6	13 (1.3)
6–11.5	776 (79.2)
> 11.5	191 (19.5)
Mean body temperature (°C)	
Mean ± SD	37.8 ± 1.4
Category of body temperature (°C)	
< 37.5	337 (34.4)
≥ 37.5	643 (65.6)
Plasmodium species	
Pf	487 (95.5)
Non-Pf (pan)	13 (4.6)
Geometric mean parasite density per μl	2921
Parasite density distribution (parasites/μl)	
< 1000	40 (7.8)
1000–10,000	120 (23.6)
> 10,000	350 (68.6)

### CareStart™ malaria HRP2/pLDH (Pf/pan) Combo test diagnostic performance

The sensitivity and specificity of CareStart™ were 99.8% [(95% CI 98.9%–99.9%) and 87.6% (95% CI 84.4%–90.3%). The positive predictive value was 89.8% (95% CI 87%–92%) while the negative predictive value was 99.8% (95% CI 98.6%–99.9%). The LR+ and LR− were 8.0 and 0.002, respectively (good diagnostic tests have LR+ > 10 and LR− < 0.1). The diagnostic accuracy for malaria was 0.5 (95% CI − 0.193 to 1.193). There was a strong agreement for the accuracy malaria diagnosis between CareStart™ and BS Microscopy (kappa = 0.863, P < 0001) (Table 2).

**Table 2:** Diagnostic performance of CareStart™ using BS microscopy as a standard method

Test	BS microscopy	
	Positive (n)	Negative (n)
CareStart™		
Positive	509	58
Negative	1	412

Measures of diagnostic performance	Value	
Sensitivity (95% CI) (%)	99.8 (98.9 to 99.9)	
Specificity (95% CI) (%)	87.6 (84.4 to 90.3)	
Positive predictive value (95% CI) (%)	89.8 (87 to 92)	
Negative predictive value 95% CI) (%)	99.8 (98.6 to 99.9)	
Diagnostic accuracy (95% CI)	0.5 (− 0.193 to 1.193)	
Likelihood ratio of a positive test	8.0	
Likelihood ratio of a negative test	0.002	
Cohen’s kappa, p value	0.863, < 0001	

## Discussion

The WHO recommends parasitological-based tests microscopy and RDT for detection of plasmodium infections at the points of care [8]. Since 2009, the Ministry of Health in Tanzania has been scaling up the use of RDT in the public health facilities [21]. However, there are several conflicting reports on diagnostic performance of RDT at the point of care leading to over or under prescribing of anti-malarial drugs [10, 15, 26].

In this study, the diagnostic performance of CareStart™ malaria HRPII/pLDH Combo test in Kibiti health centre, which has moderate malaria transmission, was evaluated. The findings show that malaria infections among the suspected patients were 57.9% and 52% as detected by RDT and microscopy respectively. The overall sensitivity and specificity of the CareStart™ was found to be 99.8% and 87.6%, respectively. These results indicate that, CareStart™ had high sensitivity with slightly lower specificity (< 90%) compared to microscopy.

CareStart™ combo performance has been evaluated using microscopy as the reference standard in different malaria transmission settings [26, 27]. High sensitivity results reported in this study are related to high parasite density (> 1000 parasites/ul) for the majority of patients tested. Previous studies have reported increase in sensitivity when parasite density was > 100 parasites/ul [27]. In addition, the findings of this study are in line with the study conducted in Ethiopia (26) which also reported good accuracy and strong agreement between RDT and light microscopy among malaria suspected patients.

In this study, the sensitivity and specificity for detection of malaria by RDT was 99.8% and 87.9% respectively. This is slightly different the overall sensitivity and specificity of 95% and 94.2%, respectively that was reported from Ethiopian patients [26] respectively Other studies have also reported high sensitivity of CareStart™ combo for detection of *P. falciparum* [14, 21, 27]. Factors such as mutation in parasite histidine-rich protein-2 reduce has been reported to be the cause the variations in diagnostic accuracy, especially sensitivity of the RDT [14, 15]. However, the results of the current study have reported higher sensitivity than the specificity.

For RDT to have good specificity during diagnosis of malaria, screening procedure, which is clinical examination, should be robust. This study used clinical malaria symptoms such as fever, general body weakness and headache as recommended in the national malaria treatment guidelines to guide diagnosis [16]. However, most of signs and symptoms for uncomplicated malaria resemble those of urinary tract infections, respiratory tract infections and gastrointestinal infections, which are common in Tanzania [3]. In this study, out of 980 individuals who were suspected to have malaria infection based on presenting symptoms, only 510 patients (52%) were confirmed to be malaria positive by microscopy. These findings support recommendations by the Tanzania Malaria Treatment Guidelines [20] emphasizing that diagnosis of malaria should be based on laboratory results using RDT and microscopy where available.

Over diagnosis of febrile infections which lead to irrational use of anti-malarial drugs has been reported in Tanzania [3], and this could be the reason for the observed low specificity compared to what has been reported elsewhere [27]. RDT accuracy is affected with the recent use of antimalarial drugs and therefore patient history of drug use is important for interpretation of RDT results [3]. In a study that was conducted in China-Myanmar malaria endemic borders, the sensitivity and specificity of CareStart™ RDT were 88.5% and 98.26%, respectively.

Another probable cause for discrepancies in diagnostic accuracy of RDT for malaria is the differences in the prevalence of *Plasmodium* species [14, 15, 27]. For instance, a study conducted in China-Myanmar malaria endemic borders reported high sensitivity of *Plasmodium vivax* (98.26%) compared to *P. falciparum* (88.52%) when using CareStart™ kit’ [27]. In the latter study, the specificity for diagnosis of *Plasmodium* species using CareStart™ kit’ was also higher for *P. vivax* (100%) compared to *P. falciparum* (90.77%). In this study, the most prevalent *Plasmodium* species was *P. falciparum* (95.5%) and, therefore, a comparison of diagnostic performance of CareStart™ RDT in individuals infected with different species of *Plasmodium* could not be made.

The diagnostic accuracy in terms of specificity (87%) results observed in this study (when using CareStart™ combo test are similar to the specificity of 84% which was reported in the study conducted in Rwanda when HRP2 + pLDH RDT was used (14). These results are an indication that RDT perform better when all of *Plasmodium* species are considered. In this study, the geometric mean parasite density was 2921 parasites/µl whereas majority of patients had parasite density greater than 10,000/µl. Although the current study did not further perform statistical analysis to establish an association between parasite density and RDT sensitivity to malaria parasites, however findings from the study which was conducted in Madagascar reported low sensitivity (60.0%) of *P. falciparum* at densities of < 100/μl and increasing sensitivity (100%) at higher parasite densities of > 500/μl [25].

In this study we compared the diagnostic performance of CareStart™ malaria HRP2/pLDH (Pf/pan) combo test in comparison with standard microscopy for detection of malaria infection among symptomatic patients. Although the results of this study have shown good performance of RDT for detection of falciparum malaria in line with the national malaria treatment guidelines [20], the use of nucleic acid-based test like PCR would have provided more evidence for comparison purposes [26, 27]. Notwithstanding this limitation, the results of this study supports continued use of RDT in areas with limited qualified human resources, electrical power supply and supportive infrastructure. Lastly this study did collect the information regarding the recent use of anti-malarials.

## Conclusion

CareStart™ malaria HRP2/pLDH (Pf/pan) had high sensitivity and strong agreement with microscopy results. However, moderate specificity of RDT resulted in substantial number of patients with false positive malaria test. It is recommended that in areas with moderate to high malaria transmission, BS microscopy should be performed to confirm RDT positive results.

## Data Availability

All data used to draw conclusion of the study is provided in the manuscript.

## Abbreviations

BS: blood smear
CI: confidence interval
DA: diagnostic accuracy
HRP-2: histidine-rich protein 2
KHC: Kibiti Health Centre
LR: likelihood ratios
MUHAS: Muhimbili University of Health and Allied Sciences
pan-pLDH: pan Plasmodium specific parasite lactate dehydrogenase
PCR: polymerase chain reaction
RDT: rapid diagnostic test
WHO: World Health Organization

## References

[1] Patouillard E, Griffin J, Bhatt S, Ghani A, Cibulskis R (2017). Global investment targets for malaria control and elimination between 2016 and 2030. BMJ Glob Health..

[2] Bhatt S, Ghani AC, Patouillard E, Cibulskis RE, Gething PW, Lynch M (2016). Potential for reduction of burden and local elimination of malaria by reducing *Plasmodium falciparum* malaria transmission: a mathematical modelling study. Lancet Infect Dis..

[3] Reyburn H, Mbatia R, Drakeley C, Carneiro I, Mwakasungula E, Mwerinde O (2004). Overdiagnosis of malaria in patients with severe febrile illness in Tanzania: a prospective study. BMJ.

[4] Amexo M, Tolhurst R, Barnish G, Bates I (2004). Malaria misdiagnosis: effects on the poor and vulnerable. Lancet.

[5] Barat L, Chipipa J, Kolczak M, Sukwa T (1999). Does the availability of blood slide microscopy for malaria at health centers improve the management of persons with fever in Zambia?. Am J Trop Med Hyg.

[6] Hamer DH, Ndhlovu M, Zurovac D, Fox M, Yeboah-Antwi K, Chanda P (2007). Improved diagnostic testing and malaria treatment practices in Zambia. JAMA.

[7] Zurovac D, Midia B, Ochola SA, English M, Snow RW (2006). Microscopy and outpatient malaria case management among older children and adults in Kenya. Trop Med Int Health..

[8] WHO (2016). Microscopy examination of thick and thin blood films for identification of malaria parasites.

[9] McMorrow ML, Aidoo M, Kachur SP (2011). Malaria rapid diagnostic tests in elimination settings-can they find the last parasite?. Clin Microbiol Infect.

[10] Tangpukdee N, Duangdee C, Wilairatana P, Krudsood S (2009). Malaria diagnosis: a brief review. Korean J Parasitol.

[11] Maltha J, Gillet P, Jacobs J (2013). Malaria rapid diagnostic tests in travel medicine. Clin Microbiol Infect.

[12] Maltha J, Gillet P, Cnops L, van den Ende J, van Esbroek M, Jacobs J (2010). Malaria rapid diagnostic tests: *Plasmodium falciparum* with high parasite densities may generate false positive *Plasmodium vivax* pLDH lines. Malar J..

[13] Maltha J, Gillet P, Jacobs J (2013). Malaria rapid diagnostic tests in endemic settings. Clin Microbiol Infect.

[14] Kozycki CT, Umulisa N, Rulisa S, Mwikarago EI, Musabyimana JP, Habimana JP (2017). False-negative malaria rapid diagnostic tests in Rwanda: impact of *Plasmodium falciparum* isolates lacking hrp2 and declining malaria transmission. Malar J..

[15] Wurtz N, Fall B, Bui K, Pascual A, Fall M, Camara C (2013). Pfhrp2 and pfhrp3 polymorphisms in *Plasmodium falciparum* isolates from Dakar, Senegal: impact on rapid malaria diagnostic tests. Malar J..

[16] Baxter R, Hastings N, Law A, Glass EJ (2008). Standard treatment guidelines and national essential medicines lists. Anim Genet.

[17] Ministry of Health [Tanzania, Mainland], Ministry of Health (MoH) [Zanzibar], National Bureau of Statistics (NBS), Government Statistician (OCGS). Tanzania Malaria Indicator Survey 2017. Dar es Salaam, 2017. https://dhsprogram.com/pubs/pdf/MIS31/MIS31.pdf.

[18] Kilonzi M, Minzi O, Mutagonda R, Sasi P, Kamuhabwa A, Aklillu E (2019). Comparison of malaria treatment outcome of generic and innovator’s anti-malarial drugs containing artemether–lumefantrine combination in the management of uncomplicated malaria amongst Tanzanian children. Malar J..

[19] Šimundić A-M (2009). Measures of diagnostic accuracy: basic definitions. EJIFCC..

[20] The United Republic of Tanzania. Standard treatment guidelines and essential medicines list. Dar es Salaam, 2013.

[21] Reyburn H, Mbakilwa H, Mwangi R, Mwerinde O, Olomi R, Drakeley C (2007). Rapid diagnostic tests compared with malaria microscopy for guiding outpatient treatment of febrile illness in Tanzania: randomised trial. BMJ.

[22] Domenica Cappellini M, Motta I (2015). Anemia in clinical practice-definition and classification: does hemoglobin change with aging?. Semin Hematol.

[23] Bwire GM, Majigo M, Makalla R, Nkinda L, Mawazo A, Mizinduko M (2019). Immunoglobulin G responses against falciparum malaria specific antigens are higher in children with homozygous sickle cell trait than those with normal hemoglobin. BMC Immunol..

[24] Florkowski CM (2008). Sensitivity, specificity, receiver-operating characteristic (ROC) curves and likelihood ratios: communicating the performance of diagnostic tests. Clin Biochem Rev..

[25] Bell D, Wongsrichanalai C, Barnwell JW (2006). Ensuring quality and access for malaria diagnosis: how can it be achieved?. Nat Rev Microbiol.

[26] Moges B, Amare B, Belyhun Y, Tekeste Z, Gizachew M, Workineh M (2012). Comparison of CareStart HRP2/pLDH COMBO rapid malaria test with light microscopy in north-west Ethiopia. Malar J..

[27] Xiaodong S, Tambo E, Chun W, Zhibin C, Yan D, Jian W (2013). Diagnostic performance of CareStart ™ malaria HRP2/pLDH (Pf/pan) combo test versus standard microscopy on falciparum and vivax malaria between China-Myanmar endemic borders. Malar J..

